# Human papillomavirus prevalence and type distribution among women attending routine gynecological examinations in Saudi Arabia

**DOI:** 10.1186/s12879-014-0643-8

**Published:** 2014-12-14

**Authors:** Abdulaziz AlObaid, Ismail A Al-Badawi, Hanan Al-Kadri, Kusuma Gopala, Walid Kandeil, Wim Quint, Murad Al-Aker, Rodrigo DeAntonio

**Affiliations:** King Fahd Medical City, Riyadh, 11525 Saudi Arabia; King Faisal Specialist Hospital & Research Centre, Riyadh, 11211, MBC-52 Saudi Arabia; Department of Obstetrics and Gynecology, King Abdulaziz Medical City, College of Medicine, King Saud bin Abdulaziz University for Health Sciences, Riyadh, 11426 Saudi Arabia; GlaxoSmithKline Pharmaceuticals Ltd, Bangalore, India; GlaxoSmithKline Vaccines, Wavre, Belgium; DDL Diagnostic Laboratory, Rijswijk, The Netherlands; Sydney Gynecologic Oncology Group, Royal Prince Alfred Hospital, Sydney, Australia

**Keywords:** Human papillomavirus, Saudi Arabia, Epidemiology

## Abstract

**Background:**

Cervical cancer (CC) is caused by persistent infection with high-risk (HR) human papillomavirus (HPV) types. In Saudi Arabia which has a population of 6.5 million women over the age of 15 years, approximately 152 new cases of CC are diagnosed and 55 women die from the disease annually. Nevertheless current epidemiological data for HPV in this population are limited. This study evaluated the prevalence and type distribution of HPV and documented the awareness of HPV infection and health-related behavior among Saudi and non-Saudi women attending routine examination.

**Methods:**

This was an observational, epidemiological cross-sectional study conducted between April 2010 and December 2011 at three hospitals in Saudi Arabia. Cervical samples from women aged ≥15 years, who were attending routine gynecological examinations were collected and tested for HPV-DNA by polymerase chain reaction and typed using the SPF_10_ DEIA/LiPA25 system. Two questionnaires on health-related behavior and awareness of HPV infection were completed.

**Results:**

A total of 417 women, mean age (standard deviation) 41.9 (±10.4) years, were included in the final analysis, of whom 77% (321/417) were Saudi nationals. HPV-DNA was detected in 9.8% women (41/417, 95% confidence interval [CI]: 7.1-13.1). The prevalence of any HR-HPV by age was: 25–34 years: 3.0%; 35–44 years: 4.5%; 45–54 years: 3.2%; >55 years: 10.9%. The most prevalent HR-HPV-types were: HPV-68/73 (5 cases); HPV-18 (4 cases); HPV-16 (3 cases). The most prevalent low risk (LR) types were HPV-6 (4 cases); HPV-42, HPV-53 and HPV-54 (2 cases each). The prevalence of HPV was higher among non-Saudi nationals vs. Saudi nationals (16.7% vs. 7.8%, *P* = 0.0234). No statistically significant risk factors were identified: 32.2% (101/314) women were aware of HPV and 89.9% (285/317) showed an interest in HPV vaccination.

**Conclusion:**

The overall prevalence of HPV was 9.8% in Saudi Arabia, but was higher in women over 55 years, as well as in non-Saudi nationals. These data provide a reference for public health authorities and may also help in determining future policies for the prevention of CC.

**Clinical trial registration:**

NCT01213459

**Electronic supplementary material:**

The online version of this article (doi:10.1186/s12879-014-0643-8) contains supplementary material, which is available to authorized users.

## Background

Cervical cancer (CC) is the third most frequent cancer in women throughout the world and was associated with an estimated 530,000 new cases and 275,000 deaths in 2008 [1]. The global age standardized incidence rate for CC is 15.2 per 100,000 population [2]. Saudi Arabia has a population of 6.5 million women over the age of 15 years [3]. Based on the available data, around 152 women are diagnosed with CC and 55 die from the disease annually, corresponding to the 11^th^ most frequent cancer among women of all ages in this population [3]. However, as not all CC cases are reported in Saudi Arabia, there is a concern that the real incidence of CC may be somewhat higher.

It is known that CC is caused by persistent infection with high-risk (HR) human papillomavirus (HPV) types [4],[5], of which HPV-16 and HPV-18 are responsible for approximately 70% of the overall cases [6]. Two HPV vaccines are currently licensed in many countries: a bivalent vaccine (*Cervarix®,* GlaxoSmithKline, Belgium) and a quadrivalent vaccine (*Gardasil®,* Merck and Co., Inc., Whitehouse Station, New Jersey), both of which are well-tolerated and have good efficacy profiles [7]-[14]. The introduction of these vaccines provides an opportunity to reduce CC, but the introduction of such a preventive measure, requires baseline data on national epidemiology and prevalent circulating HPV strains.

The epidemiology of HPV amongst women in Saudi Arabia is not fully understood and only limited publications about the prevalence, detection and genotyping of HPV [15]-[17] and attitudes towards screening are available in this population [18],[19]. In order to bridge this gap and provide baseline data, this study was undertaken to evaluate the prevalence and type distribution of HPV, including HR and low risk (LR)-types, among Saudi and non-Saudi women. The study also documented the level of awareness of HPV infection, health-related behavior, and potential risk factors for HPV infection among women attending routine gynecological screening.

## Methods

### Study design and study population

This multicenter, observational, cross-sectional, epidemiological study (NCT01213459) was conducted between April 2010 and December 2011 at three large hospitals: King Fahd Medical City (KFMC), King Faisal Specialist Hospital and Research Centre (KFSH and RC) and King Abdulaziz Medical City-National Guard Health Affairs (KAMC-NGHA) in Riyadh, Saudi Arabia. Women aged ≥15 years undergoing routine gynecological examination and willing to provide a cervical sample were enrolled. Pregnant women above 25 years or women with a known diagnosis of immunosuppression, or those who had undergone hysterectomy were excluded from the study. Cytological examination of the collected cervical samples was undertaken locally in the laboratories at each hospital. The investigator issued two questionnaires for completion by all women; these assessed health-related behavior and their awareness of HPV. The responses to these questionnaires were anonymous and confidentiality was maintained.

### Sample collection and laboratory procedures

Endocervical samples were also collected during the first visit by a trained practitioner/gynecologist using a cytobrush and placed in a liquid-based cytology transport medium (*PreservCyt®,* ThinPrep Pap Test; Cytyc Corporation, Boxborough, Massachusetts). Samples were stored at room temperature at the sites for four weeks and then at −20°C until shipment to the DDL Diagnostic Laboratory (Rijswijk, The Netherlands).

DNA was isolated from 500 μl of the cervix-vagina on a MagNA Pure Robot (Roche Diagnostics, Almere, The Netherlands) using the MagNA Pure LC Total NAILV kit and eluted in 50 μl of elution buffer [20]. Samples were tested for HPV-DNA at DDL by broad-spectrum polymerase chain reaction (PCR) using HPV short PCR fragment 10 (SPF-10) and PCR DNA enzyme immunoassay (PCR-DEIA) to amplify and recognize at least 57 HPV genotypes by hybridization with a cocktail of nine conservative probes. If positive by SPF10-DEIA the amplimers were further analyzed by Line probe assay 25 (LiPA25) version 1 system (Labo Biomedical Products, Rijswijk, The Netherlands). This Line probe assay 25 (LiPA25) version 1 system (Labo Biomedical Products, Rijswijk, The Netherlands) was used to genotype 25 HR and LR HPV types [21]. (The sequence variation within the SPF_10_ inter-primer region did not allow HPV type 68 and 73 to be distinguished [22],[23]). DEIA positive-LiPA negative samples were denoted as non-typeable.

### Sample size calculation

The primary objective of the study was to describe the prevalence and types of HPV (including multiple infections) among women ≥15 years of age. To meet this objective, an estimated HPV prevalence ranging from 10 to 30% as previously reported [17],[24],[25], was considered. Given a precision level of 0.045, the required number of subjects ranged from 188 subjects for a 10% HPV prevalence to 450 subjects for a 30% prevalence, including an assumption of 10% of subjects non-evaluable.

### Statistical analyses

The percentage of women in each category who were HPV positive was tabulated with corresponding 95% confidence intervals (CI). Descriptive analyses regarding HPV prevalence, HPV-types, age distribution, potential risk factors (education level, life-time marital partners, parity and smoking status) and HPV status were performed. An exploratory analysis was performed to assess the association between the HPV status and nationality (two sided Fisher’s exact test) and the adjusted odds ratio (adjusted for factors which are associated with the risk of HPV infection) was calculated using multivariate logistic regression model. All statistical analyses were performed using the statistical analysis software (SAS®) version 9.2.

### Ethical considerations

The study was approved by the following local ethics review bodies: Institutional Review Board at KAMC-NGHA; Institutional Review Board at KFMC; Research Ethics Committee of Office of Research Affairs at KFSH and RC. The study was conducted in accordance with the Declaration of Helsinki, good clinical practice guidelines and local rules and regulations of the country. A written informed consent was obtained from all eligible women before entering the study. The investigator communicated results as appropriate to the subjects, including the need for additional testing or treatment.

## Results

### Study population

Of 420 enrolled women, 417 were included in the final analysis (three were excluded: two due to pregnancy and one due to hysterectomy). A total of 151, 152 and 117 women were enrolled at the KFMC, KFSH and RC, and KAMC-NGHA hospitals, respectively. Overall, 319 women completed the health-related behavior questionnaire and 317 completed the HPV awareness questionnaire. The mean age (standard deviation) of the population was 41.9 (±10.45) years and 77% (321/417) were Saudi nationals. Most women (93.1%, 297/319) were married and 63.0% (201/319) had studied up to post-secondary/university level.

### Overall HPV prevalence and type distribution

HPV-DNA was detected in 41 out of 417 women (9.8%), of whom 25 had single HPV-type infection, 4 had multiple HPV-type infection and at least12 women were infected with non-typeable HPV-types. Overall, the most prevalent HR-HPV-types were HPV-68/73 (5 cases); HPV-18 (4 cases); HPV-16 (3 cases) and the most prevalent LR types were HPV-6 (4 cases); HPV-42, HPV-53 and HPV-54 (2 cases each) (Table 1).

**Table 1:** **HPV prevalence and type distribution (N = 417)**

N = 417	n	%	95% CI
**HPV negative**	376	90.2	86.9–92.9
**HPV positive**	41	9.8	7.1–13.1
Single infection	25	61.0	44.5–75.8
Multiple infection	4	9.8	2.7–23.1
Non-typeable^#^	12	29.3	16.1–45.5
HPV-types (n = 41)*			
Any high-risk HPV**	**18**	**43.9**	**28.5–60.3**
HPV-68/73	5	12.2	4.1–26.2
HPV-18	4	9.8	2.7–23.1
HPV-16	3	7.3	1.5–19.9
HPV-31	2	4.9	0.6–16.5
HPV-51	2	4.9	0.6–16.5
HPV-52	2	4.9	0.6–16.5
HPV-39	1	2.4	0.1–12.9
HPV-56	1	2.4	0.1–12.9
HPV-58	1	2.4	0.1–12.9
Any low-risk HPV***	**14**	**34.1**	**20.1–50.6**
HPV-6	4	9.8	2.7–23.1
HPV-42	2	4.9	0.6–16.5
HPV-53	2	4.9	0.6–16.5
HPV-54	2	4.9	0.6–16.5
HPV-11	1	2.4	0.1–12.9
HPV-40	1	2.4	0.1–12.9
HPV-70	1	2.4	0.1–12.9
HPV-74	1	2.4	0.1–12.9

N: number of women whose cervical samples were tested; n: number of women in a given category; 95% CI: exact 95% confidence interval.

^#^DEIA positive-LiPA negative.

*Some women were infected with multiple HR/LR HPV types.

**Includes 14 women with single HR-HPV infection and 4 women with multiple infections and at least one HR-HPV type.

***Includes 11 women with single LR-HPV infection and 3 women with multiple infections and at least one LR-HPV type.

Note: Single infection = 25/417 = 6.0% (95% CI: 3.9–8.7); Multiple infection = 4/417 = 0.9% (95% CI: 0.3–2.4); non-typeable = 12/417 = 2.9% (95% CI: 1.5–5.0).

### HPV prevalence and type distribution by age

**Figure 1:**
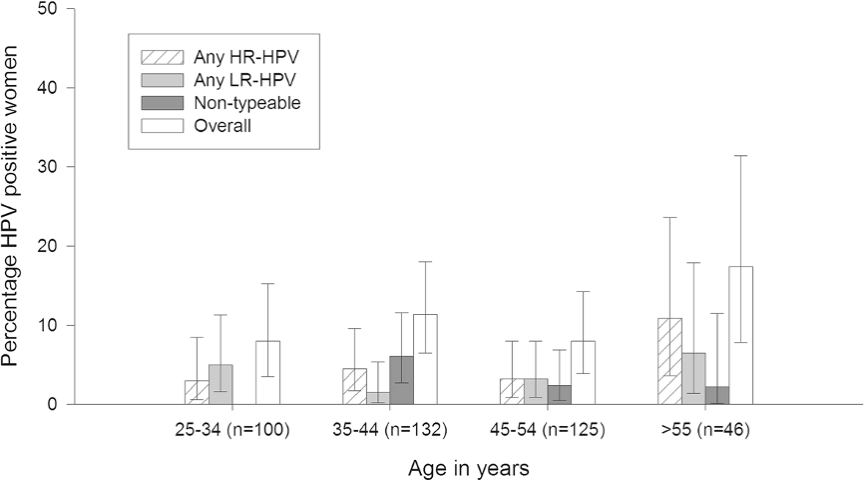
HPV prevalence and type distribution by age (N = 417*). *Note: For 2 women, dates were not available; hence age could not be estimated. Note: The error bars represent 95% confidence intervals.

The prevalence of any HR-HPV was highest (10.9%) among women over 55 years; LR-HPV-types were also found in 6.5% of this age group (Figure 1). However, no statistical significance was noted by age group.

### HPV prevalence and type distribution by nationality

**Figure 2:**
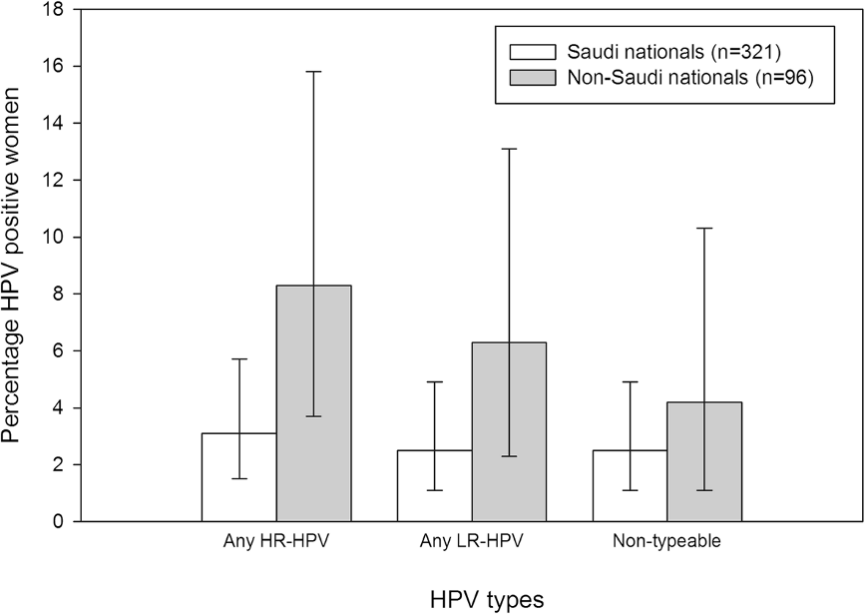
**HPV prevalence and type distribution by nationality (N = 417).** Note: The error bars represent 95% confidence intervals.

The prevalence of HPV was higher (16.7% vs. 7.8%, *P* = 0.0234) among non-Saudi nationals (n = 96) as compared with Saudi nationals (n = 321) respectively (Figure 2).

### HPV co-infection

Multiple infections were observed in four women; three of whom were infected with HR-HPV-68/73 (−68/73 with −52, −39, −53; −68/73 with −40; and −68/73 with −54). One woman had a co-infection of HR-HPV-16 with −31. No HR-HPV-18 positive women were co-infected with any other HR-HPV-types.

### Awareness and health related behavior questionnaire results

The potential risk factors of educational level, number of life-time partners, parity and smoking status assessed using univariate analyses showed no statistical associations with any HPV infection (Table 2). Of 317 women completing the HPV awareness questionnaire, 101 (32.2%) were aware of HPV and 285 (89.9%) expressed an interest in vaccination (Table 3).

**Table 2:** **Prevalence of any HPV by risk factors (N = 319)**

Risk factors	Categories	N	HPV+	%	Adj. OR	LL–UL	P value
Age at sample collection (years)	<30*	56	3	5.36	.	.	.
	30-39	124	12	9.68	3.310	0.391–28.052	0.2723
	40-49	132	12	9.09	2.097	0.230–19.085	0.5110
	50-60	89	12	13.48	4.136	0.454–37.681	0.2079
	>60	14	2	14.29	9.570	0.601–152.366	0.1097
Nationality	Non-Saudi*	96	16	16.67	.	.	.
	Saudi	321	25	7.79	0.315	0.116–0.855	0.0234
Education level	No formal education*	21	2	9.52	.	.	.
	Primary	47	3	6.38	1.039	0.144–7.489	0.9693
	Secondary	50	4	8.00	1.515	0.200–11.492	0.6879
	Post-secondary/University	201	24	11.94	1.158	0.180–7.447	0.8770
Number of marital partners	1*	286	29	10.14	.	.	.
	2-5	32	4	12.50	1.129	0.324–3.941	0.8488
Parity	0*	8	0	0.00	.	.	.
	1-2	100	11	11.00	>999.999	<0.001– > 999.999	0.9516
	3-5	112	14	12.50	>999.999	<0.001– > 999.999	0.9502
	≥ 6	71	5	7.04	>999.999	<0.001– > 999.999	0.9518
Smoking status	No*	273	30	10.99	.	.	.
	Yes	44	3	6.82	0.502	0.134–1.875	0.3053

N: number of subjects in a given cohort; %: HPV+/number of subjects with available results × 100; Adj. OR: Adjusted odds ration from simple logistic regression model Odds ratio adjusted for the other variables; 95% CI: Wald 95% confidence interval; LL: lower limit; UL: upper limit.

*Reference category.

**Table 3:** **Awareness of HPV infection among women (N = 317)**

Characteristics	Categories	n	%
How common is cervical cancer in women?	Very common	48	15.1
	Common	157	49.5
	Rare	56	17.7
	Not sure	56	17.7
What do you think is/are the main reasons for cervical cancer?*	It develops from inside	95	30.0
	Bacterial infection	49	15.5
	Viral infection	80	25.2
	None	14	4.4
	Not sure	85	26.8
Which among these can cause cervical cancer?*	Persistent infection with HPV	82	25.9
	Rous sarcoma virus	23	7.3
	Hereditary/genetic factors	115	36.3
	None	18	5.7
	Not sure	88	27.8
What do you think can turn in to cervical cancer*	Genital warts	109	34.4
	Bacterial infection	56	17.7
	Fungal infection	26	8.2
	None	23	7.3
	Not sure	108	34.1
Apart from avoiding unwanted pregnancy, what would you think can happen with using contraceptive pills*	Protects against cervical cancer	31	9.8
	Increases risk of cervical cancer	123	38.8
	No ill effect at all	77	24.3
	Not sure	86	27.1
Did you hear about HPV before?	Yes	101	32.2
	No	213	67.8
	Missing	3	-
If yes*,	General physician	28	8.8
	Friend or family member	20	6.3
	Internet	22	6.9
	TV/Magazine/Newspaper	46	14.5
	Other	14	4.4
How is HPV transmitted?*	Contaminated food/Water	10	3.2
	Mosquito bite	3	0.9
	Sexually	159	50.2
	None	20	6.3
	Not sure	127	40.1
How is cervical cancer diagnosed?*	Pap smear test	77	24.3
	Colposcopy	24	7.6
	Biopsy sample testing (histological)	122	38.5
	All above	82	25.9
	None	2	0.6
	Not sure	30	9.5
Is it possible to prevent cancer?	Yes	243	78.9
	No	26	8.4
	Not sure	39	12.7
	Missing	9	-
If yes*,	Through cancer vaccine	58	18.3
	Through responsible sexual behavior	46	14.5
	Through cervical screening	173	54.6
	Through condom use	14	4.4
If the vaccine against cervical cancer is available, would you be interested in getting vaccinated?	Yes	285	89.9
	No	32	10.1

N: number of women in a specified category for whom questionnaire data were collected.

*Women could have selected more than one option.

## Discussion

This study estimated the prevalence and type distribution of HPV in 417 women above 15 years of age attending routine gynecological screening at three large hospitals in Riyadh, Saudi Arabia. We reported a 9.8% prevalence for HPV, which is much lower than the 31.6% overall prevalence of HPV-16/18 reported previously in a small study involving subjects from Riyadh [24]. An earlier report from Jeddah reported a 5.6% prevalence of HR-types [17]. These differences could be due to many factors including differences in HPV testing technology, study size, age groups or geographical variations. The study which was undertaken in Riyadh [24] included a limited number of subjects (75 Saudi nationals and 45 from other countries) from just one hospital (which was also included in our study). The higher proportion of non-Saudi nationals in this study might help to explain the observed higher HPV prevalence [24]. Conversely, although the sample size in the study conducted in Jeddah [17] was similar to that in our present study, the exclusion of non-Saudi nationals might explain the lower reported HPV prevalence. According to available data, our study included a representative number of Saudi and non-Saudi nationals, which reflects the current demography of Saudi Arabia [25]. This might explain higher HPV prevalence in our study compared to the Jeddah study. Our study might therefore be more representative of the current situation in Saudi Arabia given the larger sample size as well as the inclusion of non-Saudi nationals.

Indeed, data from the Saudi Cancer registry suggest that there are regional differences in the percentage distribution of CC with the northern region having the highest (6.4%) percentage distribution compared with the other regions [26].

According to the United Nations classification, Saudi Arabia belongs to the Western Asia region [27], which has a lower HPV prevalence rate (2.2% [95% CI, 1.5–3.1]) compared with global rates (11.4% [95% CI, 11.3–11.5]) in women with normal cytology [2]. The crude incidence rate of CC per 100,000 women per year in Saudi Arabia is 1.3, which is lower than that in Western Asia (3.6) and the world (15.8) [2]. The prevalence of HPV in women with CC in Saudi Arabia has a broad range (43–89%) [28],[29] compared with the global prevalence (85–99%) [6]. The low rates of CC in Saudi Arabia as compared with other countries could be due to differences in sexual practices and attitudes. For example, the population in Saudi Arabia is more conservative than western countries where most of the data derive [30]. The exact reasons for these low rates in Saudi Arabia are not known, but highlight the need for recent data to better understand the disease burden of HPV and the prevalent circulating types.

The most prevalent HR-HPV-types reported in our study were HPV-68/73, 18 and 16; the most common LR-HPV-types were HPV-6, 42, 53 and 54. HPV-types 16 and 18 have been predominantly reported in HPV infections globally and the results of our study were therefore consistent [24],[31]. We did however note a high prevalence of HPV-68/73 for the first time, especially among non-Saudi nationals. HPV-68 type has been reported throughout the world, with the exception of North America, albeit at a lower prevalence [32]. However, our results should be considered with caution as only five women were positive for HPV-68/73. Future studies to substantiate this finding are therefore indicated.

Our study also found that HR-HPV infection was highest (10.9%) in the oldest age group (>55 years). These results are consistent with the general worldwide trend of higher HPV burden in older women. However, the comparator study from Bruni et al. [31] only included women with normal cytology whereas the cytology status was not known in the present study [32].

We estimated a higher proportion of infection in non-Saudi nationals compared with the Saudi nationals. This difference could be due to many reasons including different cultural behaviors such as male circumcision [32],[33], sexual behavior or prevalence in the native countries. Further studies are therefore warranted. Although, it appears that non-Saudi nationals are at a greater risk of contracting infection, prevalent HPV types indeed pose a risk of infecting Saudi nationals. Our study did not find educational level, number of lifetime partners, parity or smoking status to be significantly associated risk factors for HPV-16, HPV-18 or any HR-HPV infection.

Our study results should be interpreted with caution as the study design was cross sectional, i.e., we obtained single point estimates of women with HPV infections. These infections could have been transient and resolved on their own rather than leading to CC. In addition, since the overall number of women positive for HPV itself was low, the prevalence of HPV types should be interpreted with caution. Another limitation lies within the recruitment process: women with higher levels of education are more likely to opt for cervical cancer screening and therefore would be more likely to participate in our study. Nevertheless, our study did also include women who did not have formal education.

A major strength of this study was the high quality of HPV-DNA testing across the three hospitals in all age groups which helps to provide a representative sample of the population. The study also met the required sample size to calculate an overall prevalence of 9.8% with good precision. Furthermore, the questionnaires were completed by most enrolled women giving an important insight into behavior and attitudes, and suggesting that the introduction of a preventive measure such as vaccination would be accepted. Reports indicate that the proportion of non-Saudi nationals represent 20%–30% of the entire population in Saudi Arabia [25],[34] which is consistent with our results (23%). In addition, the proportion of women completing up to post-secondary/university level education in our study (63%) is comparable to the Organization for Economic Co-operation and Development (OECD) reports, where at least 50% of women were educated and at least 54% of women among all OECD countries completed post-secondary education [35]. These comparisons therefore indicate the representativeness of our study population.

The study results emphasize the need for a future updated policy for HPV and CC prevention in Saudi Arabia. A World Health Organization document on cancer control in the Eastern Mediterranean region describes that only 35% of CC cases present at early stage; as in other developing countries, the rest are reported at later stages when cure is unlikely, even with the best treatment [34]. It has also been reported that when women are double negative in the conventional cytological screening test and the highly sensitive HPV molecular test, then screening can be performed at longer intervals [36]. Our study findings together with these data will help determine the best strategy for targeting preventive interventions, and designing public health measures for Saudi Arabia.

## Conclusion

The overall results from this study emphasize that the HPV burden in Saudi Arabia is a cause for concern and preventive strategies such as screening, HPV-DNA testing of cervical samples and vaccination might reduce the burden of the disease. Our data can raise the awareness of local authorities and public health officials and help to guide policy in Saudi Arabia to implement strategies to prevent CC.

### Trademark

*Cervarix* is a trademark of the GlaxoSmithKline group of companies

*Gardasil* is a trademark of Merck & Co. Inc.

*PreservCyt* is a trademark of Cytyc Corporation, Massachusetts, United States of America

*Labo Biomedical Products*, Rijswijk, The Netherlands

## Authors’ original submitted files for images

Below are the links to the authors’ original submitted files for images. Authors’ original file for figure 1 Authors’ original file for figure 2

## Abbreviations

CC: Cervical cancer
CI: Confidence interval
DEIA: DNA enzyme immunoassay
HPV: Human papillomavirus
HR: High-risk
KAMC-NGHA: King Abdulaziz Medical City-National Guard Health Affairs
KFMC: King Fahd Medical City
KFSH and RC: King Faisal Specialist Hospital and Research Centre
LiPA25: Line probe assay 25
LR: Low-risk
OECD: Organization for Economic Co-operation and Development
PCR: Polymerase chain reaction
SAS: Statistical analysis software
SPF-10: Short PCR fragment 10
