# Supplementary figures and images for: Human papillomavirus prevalence and type distribution among women attending routine gynecological examinations in Saudi Arabia
Source: BMC Infect Dis. 2014 Dec 14;14:643. doi: 10.1186/s12879-014-0643-8 (PMC4272558; doi:10.1186/s12879-014-0643-8)

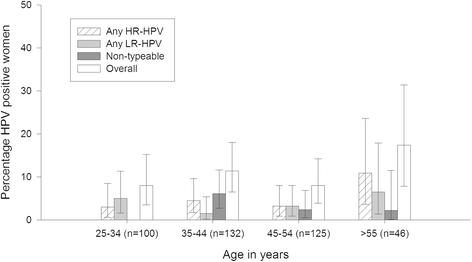

Supplement: Supplementary file 1 — Authors’ original file for figure 1 [file 12879_2014_643_MOESM1_ESM.gif]

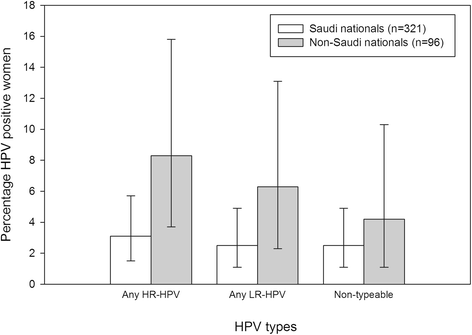

Supplement: Supplementary file 2 — Authors’ original file for figure 2 [file 12879_2014_643_MOESM2_ESM.gif]
